# Identification and analysis of micro‐exons in AP2/ERF and MADS gene families

**DOI:** 10.1002/2211-5463.12990

**Published:** 2020-11-08

**Authors:** Qi Song, Amna Bari, Huan Li, Ling‐Ling Chen

**Affiliations:** ^1^ National Key Laboratory of Crop Genetic Improvement Huazhong Agricultural University Wuhan China; ^2^ Hubei Key Laboratory of Agricultural Bioinformatics College of Informatics Huazhong Agricultural University Wuhan China

**Keywords:** AP2 domain, AP2/ERF, K‐box domain, MADS‐box, micro‐exons

## Abstract

Micro‐exons are a set of ultrashort exons with lengths ≤ 51 nucleotides. Our previous study revealed that micro‐exons were enriched in AP2 domains and K‐box domains, which are crucial components of *AP2*/*ERF* (APETALA2/ethylene‐responsive element‐binding protein) and *MADS*‐box (an acronym of MCM1, AGAMOUS, DEFICIENS and SRF) genes, respectively. In this study, we analyzed micro‐exons in the *AP2*/*ERF* family from 63 species and demonstrated that 76.8% of micro‐exons are concentrated in AP2 domains. Most micro‐exons appeared in the *AP2* subfamily of all the terrestrial plants, but not algae. In addition, micro‐exons and AP2 domains are conserved and under negative selection. The *MIKC* gene is a typical *MADS*‐box gene family in terrestrial plants and includes one MADS‐box domain and one K‐box domain. A total of 92.3% of micro‐exons were observed in K‐box domains, and two micro‐exons usually encoded a region of K‐box domain, which is the key to MADS‐box protein polymerization. Furthermore, the micro‐exons of the K‐box domain had higher ratios of nonsynonymous mutations than those of the AP2 domains. Overall, here we explored the relationships and differences among micro‐exons in *AP2*/*ERF* and *MADS* families, and revealed potential functional roles of micro‐exons in these domains.

## Graphical abstract

Here, we report that micro‐exons are enriched in AP2 domains and K‐box domains of the AP2/ERF family and MADS‐box family, respectively. Most micro‐exons are present in terrestrial plants. In addition, two micro‐exons encode a region in the K‐box domain that plays a role in polymerization. Our results illustrate the relationships and differences among micro‐exons in two transcription factor families.

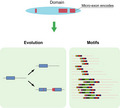

**Abbreviations**
AP2/ERF: APETALA2/ethylene‐responsive element‐binding protein
MADS: MCM1, AGAMOUS, DEFICIENS and SRF
MH63: *Oryza sativa* spp. *indica* (MH63)
NJ: Neighbor‐joining
RNA‐seq: RNA sequencing
SEP3: SEPALLATA3
SRF‐TF: SRF‐type transcription factor
TPM: transcripts per million
ZS97: *Oryza sativa* spp. *indica* (ZS97)

## Introduction

Micro‐exons are a class of exon with lengths no more than 51 nucleotides, which exist in plants, insects, mammals, etc. [1, 2, 3]. A previous study showed that the dysregulation of the splicing level of human neural‐specific micro‐exons was associated with autism [3]. In our earlier work, we systematically identified micro‐exons in rice and made a brief exploration of the structure and function of micro‐exon genes by using RNA sequencing (RNA‐seq) data [4]. However, the role of micro‐exons in plants remains unclear. It is reported that 58% of the micro‐exons in rice are enriched in functional domains, such as the AP2 domain and K‐box domain [4]. The AP2 domain and the K‐box domain are the crucial components of the APETALA2/ethylene‐responsive element‐binding protein (AP2/ERF) family and the MADS family, respectively. Both of them are key transcription factors in the ABCDE model [5]. Therefore, the study of the *AP2* */* *ERF* gene family and the *MADS* gene family is important to further comprehend the role of micro‐exons in plants.

The *AP2/ERF* gene family is a large family of transcription factors that are widely distributed in plants and regulate the spatiotemporal specific expression of genes. Recent studies have indicated that the AP2/ERF genes play a certain role in many biological processes, such as plant growth and development, biotic stress response, high salinity stress response and drought stress response [6, 7, 8]. The *AP2*/*ERF* genes mainly exist in plants, whereas genes containing AP2 domains are also found in protozoa, such as *Tetrahymena thermophila* and Apicomplexa [9, 10]. Magnani *et al*. [11] explored the origin of the *AP2*/*ERF* genes, suggesting that the *AP2*/*ERF* genes in plants were associated with HNH endonucleases in bacteria and viruses. The sequence similarity of AP2 domains between HNH endonuclease and *AP2*/*ERF* genes of plants was more than 40%. According to phylogenetic analysis, they speculated that the *AP2*/*ERF* genes of plants were derived from horizontal moving of HNH endonuclease. Supporting this hypothesis, the *AP2*/*ERF* genes were found in the green algae, but not in red algae [11]. The *AP2*/*ERF* genes have at least one AP2 domain, which is generally composed of 60 to 70 amino acids with DNA binding function. In line with the type and quantity of functional domains, they are divided into four subfamilies: ERF/DREB subfamily with only one AP2 domain, AP2 subfamily with two AP2 domains, RAV subfamily with one N‐terminal AP2 domain and one C‐terminal B3 domain, and other type of gene [7, 12]. The ERF/DREB subfamily is the largest of the AP2/ERF family containing few introns in its AP2 domain, and the AP2 subfamily is the second largest subfamily. Studies showed that the HNH endonuclease gene with an AP2 domain could be replicated and transferred in the genome like a transposon in bacteria, suggesting that *AP2*/*ERF* genes originated from intron insertion and impaired transposition/homing of the HNH endonuclease genes [11]. As for the AP2 subfamily, it suffered an extra tandem replication of AP2 domain. Generally, the AP2 domains consist of one alpha helix and three antiparallel beta sheets. These three beta sheets contact with the major groove of the DNA double helix and guide the DNA to follow the beta sheet, while the alpha helix is composed of 18 core amino acids and acts as protein‐protein interactions [13]. Recent studies have revealed that some *AP2*/*ERF* genes play crucial roles in regulation of oil biosynthesis, and some key sites are exposed by site‐directed mutation experiments [7, 14]. In addition, *AP2*/*ERF* genes are regulated by post‐transcriptional modifications, such as phosphorylation and ubiquitination [8, 15]. In contrast, some studies have demonstrated that three *AP2* subfamily genes in *Arabidopsis* play a regulatory role in the phyllotaxy development [16], *SNB* gene regulates the transformation process from spikelet meristem to floral meristem and participates in the formation of inflorescence structures [17], and *RSR1* gene regulates the synthesis of starch in rice [18].

MADS (MCM1, AGAMOUS, DEFICIENS and SRF) is abbreviated from the major members of the gene family in yeast, *Arabidopsis thaliana*, snapdragon and humans. The *MADS*‐box gene family is a class of transcription factor that exists in almost all eukaryotes, such as fungi, plants and animals [19]. However, there are only a few *MADS* genes in the fungal and animal genomes, but up to 100 in flowering plants [20]. *MADS*‐box genes play regulatory roles in plant growth and reproduction [5, 21, 22]. The typical feature of the *MADS*‐box gene family is the N‐terminal MADS‐box domain that has 56–60 amino acids. The earliest *MADS* gene was the *ARG80* identified in *Saccharomyces cerevisiae* [23]. The *MADS*‐box family is divided into two types in plants: type I (M type), which usually contains only one MADS‐box domain with DNA binding motif, is composed of a small number of exons; and type II (*MIKC* type), which includes a MADS‐box domain, an Intervening (I) domain, a Keratin‐like (K‐box) domain and a C‐terminal domain, is typically constituted of 5–8 exons [5, 24]. The *MADS* genes in plants are normally *MIKC* type, and they have conserved functions, even in specific species, some of which have acquired novel functions during evolution [5]. In general, MADS‐box proteins function as a tetramer. When MADS‐box domain plays a role in DNA binding, the K‐box domain is involved in polymerization. The micro‐exons are mainly concentrated in the K‐box domain, which has approximately 80 amino acids. Based on sequence studies, it is considered that the K‐box domain contained three segments of alpha helix, named K1, K2 and K3 [25]. The K‐box domain is a coiled‐coil structure, which might be related to protein‐protein interaction. Previous studies have illustrated that K1 and K2 are involved in DNA binding, while K3 is associated with polymerization [25, 26, 27]. The three‐dimensional structure of the K‐box domain in SEPALLATA3 (SEP3) protein had been determined by X‐ray crystal diffraction, and the results showed the K‐box consisting of two alpha helices [28]. Actually, the two assumed alpha helix structures, K2 and K3, constitute a long‐chain alpha helix, and the K‐box domain of two homologous SEP3 proteins could be joined into a tetramer by the second helix (K2 and K3). Studies have revealed that *MADS*‐box genes are involved in floral organ differentiation and responded to a variety of biotic and abiotic stresses [22]. Some genes are related to the formation of meristem and flowering time in rice, such as *OsMADS14* [29]. *OsMADS2* and *OsMADS4* genes regulate the development of stamen and petal [30], and *OsMADS25* and *OsMADS27* genes are involved in the response of osmotic stress [31].

In this study, the *AP2*/*ERF* and *MADS* genes were identified from multiple species (2 rice species and another 61 species from Ensembl Plants). Based on the species tree, we compared and analyzed the emergent time and distribution of *AP2*/*ERF* genes, *MIKC* genes and micro‐exons. The evolutionary analysis was performed on the AP2 domain and the K‐box domain sequences, respectively. We also focused on the relationship between the micro‐exons and the protein domains and explored the characteristics of the micro‐exons in the gene structures and functions. In addition, the micro‐exons were preliminary classified by the position, length and *K*_a_/*K*_s_ values of different types of micro‐exon, and their domains were calculated to understand their characteristics in evolutionary selection.

## Results

### *AP2*/*ERF* gene family in plants

A total of 10,617 *AP2*/*ERF* genes were identified from 63 plant species, including Chlorophyta, Bryophyta and Magnoliophyta. However, there was no AP2/ERF gene in three of the algae species: *Chondrus crispus*, *Galdieria sulphuraria* and *Cyanidioschyzon merolae*. In general, the *AP2*/*ERF* genes are classified into four subfamilies: *AP2* subfamily, *ERF*/*DREB* subfamily, *RAV* subfamily and others. Among the 60 species with *AP2*/*ERF* genes, the *ERF*/*DREB* subfamily and the *AP2* subfamily are the first two largest subfamilies, respectively (Table S1). Furthermore, we also observed that 76.8% of the micro‐exon regions overlapped with AP2 domains, whereas other regions outside the domains have only a few micro‐exons (Table S2), suggesting that the micro‐exons may be associated with the function of the AP2 domain.

**Fig. 1:**
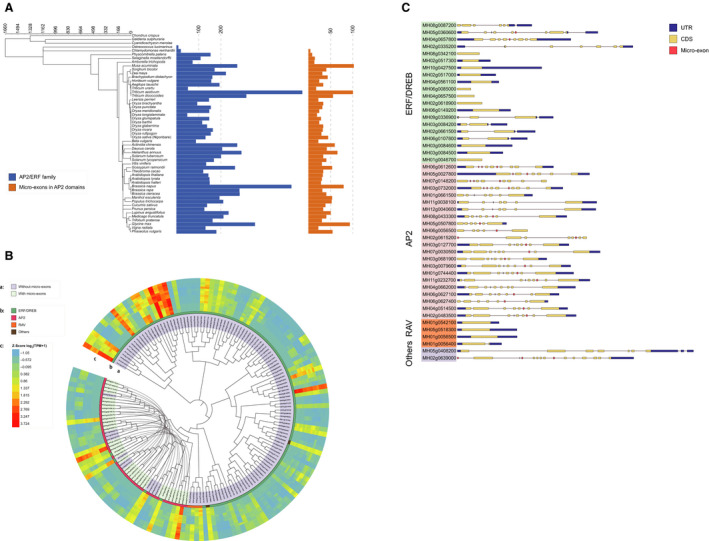
Overview of *AP2*/*ERF* genes in different plants. (A) Identified *AP2*/*ERF* genes and micro‐exons in 53 plant species. Blue color represents the number of *AP2*/*ERF* genes, and orange color represents the number of micro‐exons in AP2 domains. (B) Clustering analysis of *AP2*/*ERF* genes in MH63 based on AP2 domains. The inside circle illustrates *AP2*/*ERF* genes with or without micro‐exons, and the middle circle illustrates four subfamily types of *AP2*/*ERF* gene. The outside circle illustrates the gene expression level, the *Z* score of log_2_(TPM + 1). From inside to outside, it represents gene expression in flag leaf, panicle, root and shoot, in high and low temperature and in long and short daytime. (C) Gene structures of *AP2*/*ERF* genes in MH63. Forty‐eight *AP2*/*ERF* genes from four subfamilies are shown by different colors; blue, yellow and red indicate UTRs, CDSs and micro‐exons, respectively.

We observed that there are 19 and 8 *AP2*/*ERF* genes in algae *Chlamydomonas reinhardtii* and *Ostreococcus lucimarinus*, respectively, whereas the other species generally contained 100–300 *AP2*/*ERF* genes, indicating that the *AP2*/*ERF* genes might appear during the differentiation of the algae ancestors (about 1.66 billion years ago), and the number of genes grew rapidly in terrestrial plants (about 1.16 billion years ago) (\* MERGEFORMAT; Fig. 1A). *Triticum aestivum*, *Triticum dicoccoides* and *Brassica napus* embodied 566, 314 and 517 *AP2*/*ERF* genes separately because of the different numbers of their polyploid genomes (hexaploid, tetraploid and tetraploid, respectively). Besides, some diploid species with normal genome size had a large number of *AP2*/*ERF* genes, such as *Musa acuminata* and *Glycine max*. On the whole, approximately 13% of the *AP2*/*ERF* genes contained micro‐exons. Among the five algae species, only *Chlamydomonas reinhardtii* had six micro‐exons in their AP2 domains of *AP2*/*ERF* genes (\* MERGEFORMAT; Fig. 1A).

### Phylogenetic analysis of *AP2*/*ERF* genes

To understand the characteristic of *AP2*/*ERF* genes in rice, we extracted the AP2 domain sequences from all of the *AP2*/*ERF* genes of *Oryza sativa* spp. *indica* (MH63) for phylogenetic analyses. Considering that the *AP2* subfamily genes have two AP2 domains, they are labeled as R1 and R2, respectively, according to the position from the N terminus to C terminus. In total, MH63 had a total of 141 *AP2*/*ERF* genes, including 113 *ERF*/*DREB*, 22 *AP2*, 4 *RAV* and 2 other types of *AP2*/*ERF* subfamily gene. Like most plant species, the *ERF*/*DREB* subfamily had the largest number of *AP2*/*ERF* genes. The *AP2* subfamily was the second largest, whose domains R1 and R2 were clustered separately on the phylogenetic tree (Fig. 1B). But these two domains were still relatively close in evolution compared with the AP2 domains of other subfamilies. Besides the AP2 domains of two *ERF*/*DREB* genes that had micro‐exons, the other micro‐exons were in the two AP2 domains of the *AP2* subfamily genes, which might indicate a relationship between the micro‐exons and the *AP2* subfamily.

Based on the expression heatmap by RNA‐seq data of MH63 and *Oryza sativa* spp. *indica* (ZS97), some genes in each subfamily had relatively higher expression levels (Fig. 1B). Besides, the *AP2*/*ERF* genes were in tissue‐specific expression , while the changes in temperature or light conditions had a marginal effect on the gene expression. In the phylogenetic tree, the AP2 domains of four *ERF*/*DREB* subfamily genes were clustered together with the AP2 domains of the *AP2* subfamily genes, while the RAV subfamily genes were aggregated with other *ERF*/*DREB* subfamily genes. These results demonstrated that the AP2 domains of the *ERF*/*DREB* gene had the sequence diversity and also had similar structures to the AP2 domains of the RAV subfamily and the *AP2* subfamily separately.

During further study for exploring and comparing the gene structures of *AP2*/*ERF* genes, we selected 20 *ERF*/*DREB* subfamily genes, 22 *AP2* subfamily genes, 4 *RAV* subfamily genes and 2 other types of *AP2*/*ERF* gene from MH63 as examples (Fig. 1C). This showed that the composition of the *ERF*/*DREB* subfamily genes was diverse, which included single‐exon genes, genes consisting of several long exons and genes containing multiple short exons. However, the *ERF*/*DREB* subfamily genes contained few micro‐exons. Additionally, the *AP2* subfamily genes were more consistent in composition than other subfamilies, and they had multiple short exons and generally included one to two micro‐exons. Furthermore, three *RAV* subfamily genes were single‐exon genes, whereas the other one had two long exons. The coding sequence (CDS) regions of these four genes were all in a single exon. It is worth mentioning that the two *AP2* subfamily genes, MH06g612600 and MH05g27800 (*RSR1*), had similar exon composition, but MH06g612600 had two micro‐exons in tandem, while MH05g27800 had one micro‐exon and one longer exon in the same position. Consistent with this result, ZS97 also had these genes.

**Fig. 2:**
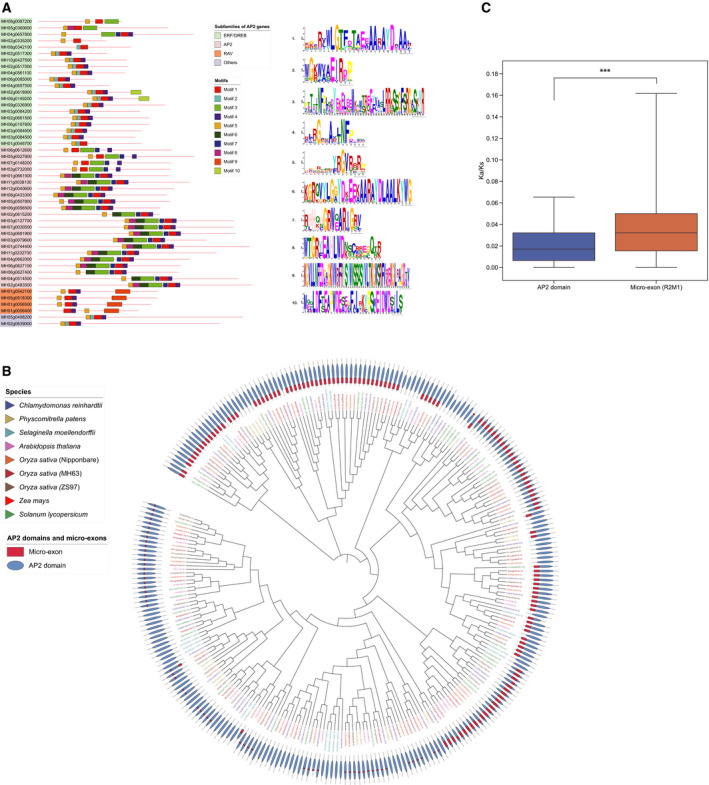
The analyses of micro‐exons and the *AP2* subfamily. (A) Motif analysis of *AP2*/*ERF* genes in MH63. The genes from the 4 subfamilies are marked in 4 different colors, and 10 motifs are marked in 10 different colors. (B) Phylogenetic analysis of the *AP2* subfamily among nine species based on AP2 domains. The inside circle illustrates *AP2*/*ERF* genes in nine species, and the outside circle illustrates micro‐exon regions in AP2 domains. (C) Comparison of *K*_a_/*K*_s_ values between micro‐exons and AP2 domains. *** *P* <0.001. The blue color represents *K*_a_/*K*_s_ values of AP2 domains, and the orange color represents *K*_a_/*K*_s_ values of micro‐exons (R2M1), Wilcoxon signed‐rank test, one‐sided, *n* = 266, *P* < 0.001.

We then identified 10 motifs from all of the *AP2*/*ERF* genes of MH63, and some genes of four subfamilies were selected to display and compare (Fig. 2A). The results declared that most of the *ERF*/*DREB* subfamily genes contained a tandem combination (Motif 5, Motif 2, Motif 1 and Motif 4), which covered the AP2 domain. The genes not containing this motif combination were closer to the *AP2* subfamily in evolution. In addition, the *AP2* subfamily genes typically had a seven‐motif tandem. The first three motifs (Motif 5, Motif 8 and Motif 6) covered the first AP2 domain (R1), while the second three motifs (Motif 7, Motif 1 and Motif 4) covered the other (R2), and Motif 3 was in the spacer between the two AP2 domains. We also observed that R1 had the same Motif 5 as the one in the ERF/DREB subfamily, and R2 had the same combination of Motif 1 and Motif 4 as that of the ERF/DREB subfamily, suggesting that R1 and R2 might have the uniform origin as the AP2 domain of the ERF/DREB subfamily. This is in accordance with a previous evolutionary model of *AP2*/*ERF* genes in plants [11]. Furthermore, the *RAV* subfamily genes also had the same combination of Motif 5, Motif 1 and Motif 4, while the two other types of genes had the same motif combination as the AP2 domain of the ERF/DREB subfamily.

For further study of the relationship between *AP2* subfamily genes and micro‐exons, we collected 134 *AP2* subfamily genes from MH63, ZS97 and other seven species for phylogenetic analysis. The results revealed that most of the R1 and R2 domains in different species were clustered explicitly on the phylogenetic tree, respectively (Fig. 2B). Meanwhile, some R1 domains of *Chlamydomonas reinhardtii*, *Zea may* and *Solanum lycopersicum* were in a block with the R2 domain of other species, and no micro‐exons were found in these domains, reflecting a close evolutionary relationship.

### Micro‐exons in AP2 domains

According to the position and length of the micro‐exons in the AP2 domains, there are three main types of micro‐exon in the R1 domain, which are 9, 26 and 31 bp and labeled as R1M1, R1M2 and R1M3, respectively. There is only a 45‐bp micro‐exon in the R2 domain, labeled as R2M1.

The micro‐exons in the R1 domain had smaller length than that in the R2 domain, and the R1M1 appeared only in the R1 domain. The tandem of R1M2 and R1M3 and the R2M1 were generally paired in the same type of *AP2* subfamily gene. Hence these genes could have analogical structures and functions. Besides, there was another type of micro‐exon located in the upstream boundary of the R2 domains, and it widely existed in many species, such as *Physcomitrella chinensis*.

In fact, we discovered that the amino acid sequences of R1M1 were highly conserved among multiple species, and their corresponding nucleic acid sequences were also consistent in lengths and positions in their coding frame (Fig. S1). However, these sequences had several synonymous mutations among the species, suggesting that R1M1 was under a strong purification selection during evolution. Moreover, the amino acid sequence VYLG of R1M1 was also conserved in R1M2 and R2M1, and tyrosine (Y) was predicted as a DNA binding site [32]. It might reveal the role and significance of the micro‐exons in their conserved sequences and functions of the AP2 domain. In addition, R1M2, R1M3 and R2M1 were merely found in *Arabidopsis*, rice, maize and tomato, declaring that these micro‐exons may be generated in a relatively late period. It was also found that such genes had high expression levels, such as *RSR1*, a transcriptional factor regulating starch synthesis in rice.

We then calculated and compared the *K*_a_/*K*_s_ values of the micro‐exons and their AP2 domain sequences. Generally, it is believed that *K*_a_/*K*_s_ > 1 means positive selection, *K*_a_/*K*_s_ = 1 means neutral selection and *K*_a_/*K*_s_ < 1 means purification selection. We observed that all of the *K*_a_/*K*_s_ values of the four types of micro‐exon and AP2 domains were less than 1, indicating that all of them were under purification selection. Furthermore, the sequences of R1M1, R1M2 and R1M3 mainly represented synonymous mutations or complete uniformity among different species, illustrating that the micro‐exons in the R1 domain were also under strong purification selection. In contrast, the *K*_a_/*K*_s_ values of R2M1 were slightly higher than that of the AP2 domains. It demonstrated higher ratios of nonsynonymous mutations occurred in R2M1 than that of the AP2 domains (Fig. 2C, Wilcoxon signed‐rank test, one‐sided, *n* = 266, *P* < 0.001).

### *MADS*‐box genes in plants

**Fig. 3:**
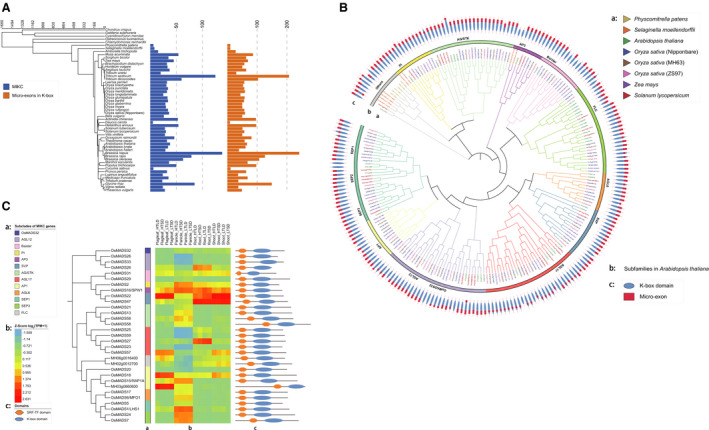
The analyses of *MIKC* genes in plants. (A) The overview of *MIKC* genes and micro‐exons in 53 species. Blue color represents the number of *MIKC* genes, and orange color represents the number of micro‐exons in K‐box domains. (B) Phylogenetic analysis of *MIKC* genes among eight species based on K‐box domains. The inside circle illustrates *MIKC* genes among eight species, the middle circle illustrates 15 *MIKC* subfamilies and the outside circle illustrates the location of micro‐exons and K‐box domains. (C) Gene expressions and domains in *MIKC* genes of MH63; the left column demonstrates 15 *MIKC* subfamilies, the middle heatmap represents gene expression levels with the *Z* score of log_2_(TPM + 1) and the right chart illustrates the position of MADS‐box domain and K‐box domain in *MIKC* genes.

Apart from the analysis of *AP2*/*ERF* genes, we also identified 2122 *MIKC* types of *MADS* gene from all of the 63 species and found that most species had 20–60 *MIKC* genes. Among the five algae species (*Chlamydomonas reinhardtii*, *Chondrus crispus*, *Cyanidioschyzon merolae*, *Galdieria sulphuraria*, *Ostreococcus lucimarinus*), no *MIKC* genes were recognized. In agreement with the results of the *AP2*/*ERF* family, the *MIKC* subfamily genes were also concentrated in *Triticum aestivum* (125 genes, hexaploid), *Triticum dicoccoides* (60 genes, tetraploid) and *Brassica napus* (138 genes, tetraploid) because of their polyploid genomes. Additionally, some diploid species, such as *Glycine max* (85 genes) and *Brassica rapa* (79 genes), also had an enormous number of the *MIKC* genes. However, in terrestrial plants, *Cucumis sativus* and *Daucus carota* had quite a small number of *MIKC* genes, which had only six and five genes, respectively. Combining the species tree of 53 species, the results suggested that the *MIKC* genes might originate from the ancestor of terrestrial plants about 532 million years ago (Fig. 3A). Besides, most K‐box domains of the *MIKC* genes contained two micro‐exons, so the ratio of micro‐exons to *MIKC* genes in each species was close to 2 : 1. The micro‐exons of the *MIKC* genes (92.3%) were enriched in the K‐box domain, but only a few micro‐exons were outside the K‐box domains (Table S3).

### Phylogenetic analysis of *MADS*‐box genes

To investigate the evolutionary relationship between the micro‐exons and the MIKC subfamily genes, we collected the sequences of K‐box domain from eight species to construct a phylogenetic tree. The phylogenetic tree illustrated that the *MIKC* genes of *Physcomitrella patens* and *Selaginella moellendorffii* were less abundant and accumulated only in several clusters, whereas the *MIKC* genes of the other six species were evenly distributed (Fig. 3B). This result indicated that the *MIKC* genes of these six species had extensive categories and functions. Based on the gene symbol of *Arabidopsis* and rice, the *MIKC* genes were classified into 15 major subfamilies: *SEPALLATA1* (*SEP1*), *SEP2*, *SEP3*, *AP1*, *AP3*, *OsMADS32*, *AGAMOUS‐LIKE6 (AGL6)*, *AGL12*, *AGL17*, *SHORT VEGETATIVE PHASE* (*SVP*), *FLOWERING LOCUS C* (*FLC*), *Bsister*, *AGAMOUS* (*AG*)/*SEEDSTICK* (*STK*), *PISTILLATA (PI)* and a class of undefined genes (Table 1). Most subfamilies had two tandem micro‐exons at the C terminus of the K‐box domain, except *AGL17*, which had only one micro‐exon at its corresponding position. Additionally, there were also two micro‐exons in the N terminus of the *MIKC* genes. Two types of *MIKC* genes existed in *Physcomitrella patens*, including K‐box domains with two micro‐exons and without micro‐exons.

**Table 1:** *MIKC* genes of MH63 and the corresponding gene symbols.

Gene ID in MH63	CGSNL gene symbol	TAIR gene symbol
*MH01g0115100*	*OsMADS58*	*AG/STK*
*MH01g0577700*	*OsMADS32*	*OsMADS32*
*MH01g0730200*	*OsMADS2*	*PI*
*MH01g0733300*	*OsMADS21*	*AG/STK*
*MH02g0012700*	*–*	*–*
*MH02g0074400*	*OsMADS29*	*ABS*
*MH02g0443900*	*OsMADS27*	*AGL17*
*MH02g0546200*	*OsMADS6/MFO1*	*AGL6*
*MH02g0600000*	*OsMADS57*	*AGL17*
*MH02g0626300*	*OsMADS22*	*SVP*
*MH03g0087600*	*OsMADS47*	*SVP*
*MH03g0114800*	*OsMADS1/LHS1*	*SEP1*
*MH03g0660600*	*–*	*–*
*MH04g0256600*	*OsMADS25*	*AGL17*
*MH04g0449000*	*OsMADS26*	*AGL12*
*MH04g0595500*	*OsMADS17*	*AGL6*
*MH04g0626000*	*OsMADS31*	*ABS*
*MH05g0106500*	*OsMADS58*	*AG/STK*
*MH06g0016400*	*–*	*–*
*MH06g0081500*	*OsMADS5*	*SEP1*
*MH06g0310100*	*OsMADS59*	*AGL17*
*MH06g0697000*	*OsMADS16/SPW1*	*AP3*
*MH07g0016900*	*OsMADS15/RAP1A*	*AP1*
*MH07g0474300*	*OsMADS18*	*AP1*
*MH08g0018300*	*OsMADS26*	*AGL12*
*MH08g0423400*	*OsMADS23*	*AGL17*
*MH08g0528700*	*OsMADS7*	*SEP3*
*MH09g0414200*	*OsMADS24*	*SEP3*
*MH12g0114200*	*OsMADS33*	*AGL12*
*MH12g0114400*	*OsMADS13*	*AG/STK*
*MH12g0325000*	*OsMADS20*	*AP1*

We further focused on the *MIKC* genes in MH63. Based on the genomic annotation, we found that the *MIKC* genes typically contained five to nine exons and one to three micro‐exons, most of which had various lengths of UTRs (Fig. S2). In addition, the expressions of *MIKC* genes were tissue specific, whereas the expression differences were not significant under different daytime or temperature conditions (Fig. 3C). The genes in the same subfamily generally had a similar expression pattern. Besides, we constructed two Neighbor‐joining (NJ) trees of MH63 based on MADS‐box and K‐box domains separately, demonstrating that the genes from the equal subfamily were clustered together in both trees. However, we also observed that the clade of *OsMADS32* was close to that of *AGL12* in the tree of K‐box domains, but not in the tree of MADS‐box domains (Fig. 3C and Fig. S3).

### Micro‐exons in K‐box domains

**Fig. 4:**
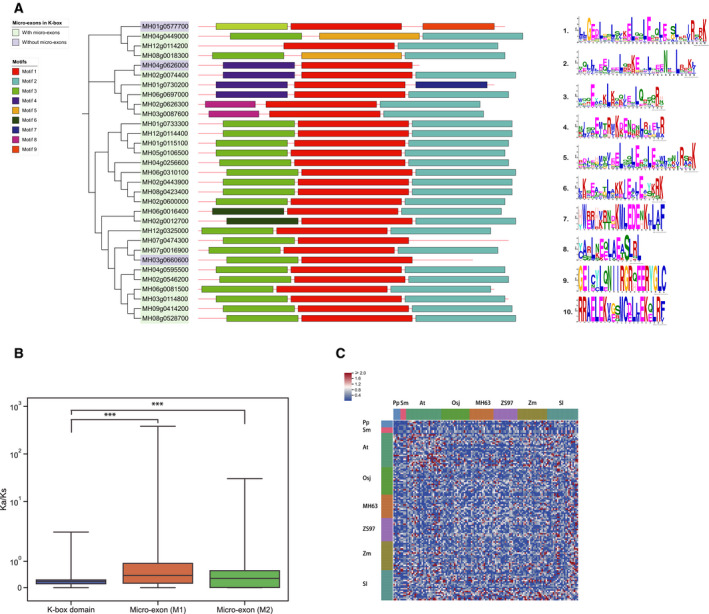
The analyses of the micro‐exons and K‐box domains. (A) The Motif analysis of K‐box domains in MH63. The left tree demonstrates the genes with or without micro‐exons, the middle strips demonstrate different motifs in K‐box domains, and the right chart demonstrates the 10 motifs. (B) Comparison of *K*_a_/*K*_s_ values between micro‐exons and K‐box domains. ***P < 0.001. The blue color represents *K*_a_/*K*_s_ values of K‐box domains, the orange color represents *K*_a_/*K*_s_ values of micro‐exon (M1), and the green color represents *K*_a_/*K*_s_ values of micro‐exon (M2); Wilcoxon signed‐rank test, one‐sided, *n* = 8515, M1 and the K‐box domains: *P* < 0.001, M2 and the K‐box domain: *P* < 0.001. (C) The heatmap of *K*_a_/*K*_s_ values of micro‐exons (M2) among eight species. The two strips illustrate eight species in different colors. The red color represents *K*_a_/*K*_s_ ≥ 1, the white color represents *K*_a_/*K*_s_ = 1 and the blue color represents *K*_a_/*K*_s_ ≤ 1.

To determine the composition of the K‐box domains, we obtained 10 conserved motifs of the K‐box domain from the eight species by using MEME. Nine of these motifs were found in MH63 (Fig. 4A). The K‐box domains were normally composed of three motifs in tandem. Moreover, the K‐box domains could be further classified based on the various motifs. Motif 1 was more conserved in several types, and the Motif 2 mainly embodied Motif 1. But two genes of the K‐box domain have Motif 5, suggesting that Motif 5 is similar to Motif 1. In addition, Motif 2 mostly made up the third motif of the K‐box domains (Motif 2, NQLLLEZIEELQRKEQLLQEENKDLRRKL). This motif was present in approximately 73% of the K‐box domains and consisted of exactly two micro‐exons in tandem. Previous research has revealed that this region is the key to the polymerization of MADS‐box proteins. Therefore, the sequences of the two micro‐exons may be closely related to polymerization.

In addition, we calculated the *K*_a_/*K*_s_ values of the two major types of micro‐exon and the K‐box domains in the eight species. The results showed that the K‐box domains had lower *K*_a_/*K*_s_ values than those of the two types of micro‐exon (Fig. 4B). We speculated that higher ratio of nonsynonymous mutations was in the micro‐exons than that of the K‐box domains. In particular, the *K*_a_/*K*_s_ values of the micro‐exons in some gene pairs were even higher than 1, indicating a strong positive selection in evolution. Besides, the results also revealed that the micro‐exons of *Physcomitrella patens* and *Selaginella moellendorffii* possessed relatively low *K*_a_/*K*_s_ values, whereas many micro‐exons of *Arabidopsis thaliana* had rather high values (Fig. 4C).

## Discussion

Our previous study revealed that micro‐exons are concentrated in AP2 domains and K‐box domains, corresponding to the *AP2*/*ERF* family and *MADS* family genes [4]. These genes are rarely found in algae but wildly distribute in terrestrial plants. Some species have a large number of genes because of polyploidization.

AP2 subfamily genes are one type of *AP2*/*ERF* gene containing two AP2 domains (R1 and R2). R1 clustered with R2 in some species on the phylogenetic tree, proclaiming the relationship between R1 and R2. The motif analysis of *AP2*/*ERF* genes also suggested that the two AP2 domains might derive from a single AP2 domain, consistent with a previous evolutionary model [11]. Most micro‐exons existed in *AP2* subfamily genes, although there were also *AP2* subfamily genes without micro‐exons, especially in primitive species, such as moss. This indicated that micro‐exons might emerge after the duplication of the AP2 domain. There were several kinds of micro‐exon in the two AP2 domains of *AP2* subfamily genes. Some micro‐exons were widely distributed among terrestrial plants, whereas other micro‐exons were restricted to some specific species, such as rice, maize and tomato. It is speculated that micro‐exons might appear in different periods or they would lose in some species. Further study showed that AP2 domains and micro‐exons were highly conserved, and the nucleotide sequences of micro‐exons were more conserved than that of AP2 domains. The results also revealed that micro‐exons in R2 had a relatively higher ratio of nonsynonymous mutations than that of R1. Therefore, we considered that the mutations of micro‐exons in R2 might relate to adaption of molecular function in various *AP2* subfamily genes.

*WRI1*, an *AP2* subfamily gene, is a key regulator of fatty acid synthesis. Also, the orthologs of *WRI1* reveal a conserved 9‐bp micro‐exon encoding the amino acids VYL [14]. Furthermore, Ji *et al*. [33] have demonstrated that there are two isoforms of *RcWRI1* in castor bean, one of which lacks three amino acid residues VYL encoded by a 9‐bp micro‐exon, and it appears to be more active than the other one. Actually, it has been reported that four *WRI1* genes (*OsWRI1‐1*, *OsWRI1‐2*, *OsWRI3* and *OsWRI4*) are found in Nipponbare. The *OsWRI1‐1* gene contains GCL, while the *OsWRI1‐2* gene contains VYL, which is encoded by a micro‐exon [34]. The high activity of *OsWRI1‐1* is verified in *Arabidopsis*, and amino acid replacement or deletion would reduce its activity. We found three *WRI1* genes in MH63/ZS97, except *OsWRI1‐1*. It may be the difference between these two subspecies, *indica* and *japonica*, and this difference might lead to different regulations in fatty acid synthesis.

*MIKC* genes are typical *MADS*‐box genes in plants, which contain a MADS‐box and a K‐box domain, and micro‐exons were concentrated on the K‐box domains. Among eight selected species, the genes of *Physcomitrella patens* occurred in only one subclade (means have one subfamily gene), whereas the genes from other species generally contained several subfamilies, indicating that the *MIKC* genes might get a rapid differentiation both in sequences and functions. Previous studies have reported that these genes are crucial regulators of gametophytic and embryo development in plants; for instance, *AGL80* and *AGL61* genes could control the differentiation of the central cell by forming a protein dimer in *Arabidopsis thaliana* [5]. Besides, we compared the phylogenetic trees of MH63 based on MADS‐box and K‐box domains, and found that all of the subclades were consistent, suggesting a good correspondence of these two domains. In addition, MIKC proteins function as a tetramer, and K‐box domain is related to polymerization. Our study discovered that K‐box domains were comprised of three motifs, and the third one (K3) was the key role in polymerization [25]. Also, K3 had two micro‐exons, suggesting that these two micro‐exons might carry out this function together. Furthermore, these two micro‐exons had a significantly higher ratio of nonsynonymous mutations than that of the K‐box domains, reflecting that micro‐exons were under positive selection among part gene pairs of Angiospermae. It can be considered that the micro‐exons might gain broad sequence diversity among Angiospermae and relate to the novel functions. Further research is required to understand the relationship between various functions and the micro‐exon sequences.

Overall, we explored micro‐exons in the two related gene families and revealed the relationship between micro‐exons and domains in function and evolution. We then confirmed that the micro‐exons were enriched in the functional domains in plants, and they might occur in different stages. Some types of micro‐exon were highly conserved as the domains, while some others had high ratios of nonsynonymous mutations, which might adapt to the gene diversity. This work will facilitate the study of micro‐exons in plants and enhance our understanding of the relationship between micro‐exons and functional domains.

## Materials and methods

### Data collection

We collected all of the protein sequences and genomic annotations of MH63 and ZS97 from Rice Information GateWay (RIGW, http://rice.hzau.edu.cn/rice_rs1; version RS1; accessed on July 4, 2016). Furthermore, the sequences and genomic annotations of another 61 species from Ensembl Plants (http://plants.ensembl.org; Release 43) were downloaded. The RNA‐seq data of MH63 and ZS97 were obtained from our laboratory and used for gene expression analysis [Illumina HiSeq 2000 Wuhan, Hubei province, China, 101‐nucleotide reads, pair end; four tissues (flag leaf, panicle, seedling shoot and root)] under different conditions: high temperature and long day, high temperature and short day, low temperature and long day and low temperature and short day. Each one had two replicates: high temperature: 28–32 °C; low temperature: 22–25 °C; long day: 14 h light and 10 h dark; short day: 10 h light and 14 h dark.

### Identification of *AP2*/*ERF* and *MADS*‐box genes

First, we collected all of the protein sequences in MH63, ZS97 and another 61 species. As to the gene with multiple transcripts, the longest protein sequence was chosen. Then InterProScan was applied to search domains in the sequences based on the Pfam database [32]. Depending on different domains, the detailed methods were as follows.

*AP2*/*ERF* genes are a set of genes with AP2 domains. The proteins with AP2 domains (PF00876) were identified in all species. Generally, the *AP2*/*ERF* gene family can be divided into four subfamilies in line with the number of AP2 domains and some other domains: ERF/DREB subfamily with only one AP2 domain, AP2 subfamily with two AP2 domains, RAV subfamily with one AP2 domain and one B3 domain, and the others with one AP2 domain and other domains. Then the identified genes were classified into the above four types of subfamily.

*MADS*‐box genes usually contain a MADS‐box domain and a K‐box domain in plants, which is known as the MIKC subfamily. Actually, our previous research revealed that micro‐exons are mainly enriched in K‐box domains. Hence this study focused on the *MIKC* genes in plants. As far as we know, the MADS‐box domain is recorded as SRF‐type transcription factor (SRF‐TF, PF00319) in the Pfam database. The protein sequences containing one SRF‐TF domain and one K‐box domain (PF01486) were retained, and these genes were regarded as candidate *MIKC* genes.

The last was the graphic display. According to the species names of Ensembl Plants, we got 53 species tree and divergence times from TimeTree (http://timetree.org/). Then we annotated the species tree by the number of the genes and micro‐exons in the domains.

### Phylogenetic analyses of the two gene families

We constructed a phylogenetic tree of the *AP2*/*ERF* genes through all the AP2 domain sequences of MH63. For the *AP2* subfamily, the two AP2 domains were labeled as R1 and R2 according to the order from the N terminus to the C terminus. The sequences of all of the AP2/ERF genes in MH63 were integrated into one file and aligned by using Clustal Ω with the default parameters [35]. Based on the aligned results, MAGE‐CC (7.0.26) was applied to construct the NJ tree by the Jones‐Taylor‐Thornton model with 2000 bootstraps [36, 37]. Then iTOL (https://itol.embl.de/) was used to annotate the phylogenetic tree [38]. Every gene in the tree was annotated by the expression level, subfamily classification and presence/absence of the micro‐exons. To obtain the expression level of the AP2/ERF genes in different tissues and conditions, we used HISAT2 [39] to map the RNA‐seq data of MH63 to its genome and used StringTie [39, 40, 41] to assemble and generate the information of transcriptome abundance based on the genome annotation. The gene abundances [transcripts per million (TPM)] were also calculated by StringTie. Because micro‐exons were concentrated on AP2 domains of the AP2 subfamily, we gathered and analyzed the AP2 domain sequences in nine species (MH63 and ZS97, *Chlamydomonas reinhardtii*, *Physcomitrella patens*, *Selaginella moellendorffii*, *Arabidopsis thaliana*, *Solanum lycopersicum*, *Oryza sativa* spp. *Japonica* and *Zea mays*) for further studying the relationship between the AP2 domains and the micro‐exons. The genes of these nine species were labeled by different colors, and the locations of the micro‐exons in the AP2 domains were also marked in the phylogenetic tree. In addition, selected *ERF*/*DREB* subfamily genes (20 genes), all of the *AP2* subfamily genes (22 genes), all of the *RAV* subfamily genes (4 genes) and other *AP2*/*EFR* genes (2 genes) were chosen to display the gene structures based on genomic annotations, of which the UTRs, CDSs and micro‐exons were marked. Besides, we also collected the sequences of all the *AP2*/*ERF* genes in MH63 and predicted their motifs using MEME (5.0.5). The parameter of the motif numbers was set to 10, and the length was set to 6–50 amino acids [42].

As for *MIKC* genes, eight species (MH63 and ZS97, *Physcomitrella patens*, *Selaginella moellendorffii*, *Arabidopsis thaliana*, *Solanum lycopersicum*, *Oryza sativa* spp. *japonica* and *Zea mays*) were applied for evolutionary analysis. K‐box domain sequences were collected and used to construct a NJ tree with Jones‐Taylor‐Thornton model by mega‐cc. The genes and the positions of the micro‐exons in the K‐box domains were displayed. In addition, the *MIKC* genes were divided into 15 subfamilies in line with the subfamilies in *Arabidopsis* [43]. *OsMADS32* was considered as an exceptional subfamily, which can be distinguished from the genes in *Arabidopsis*. Then the absence of *MIKC* genes in *Arabidopsis* and rice were treated as other undefined genes. Furthermore, we also extracted the sequences of K‐box domains and MADS‐box domains in MH63 to construct the NJ trees for understanding the expression levels of *MIKC* subfamily genes under different conditions. Also, all of the *MIKC* genes in MH63 were labeled as CGSNL gene symbols and the 15 subfamilies. In addition, the gene structures of all 31 *MIKC* genes in MH63 were also predicted and shown.

### Analyses of micro‐exons in the domains

We chose four major types of micro‐exon in AP2 domains for further analysis. Three were in the R1 domain labeled as R1M1, R1M2 and R1M3, respectively, and the remaining one was in the R2 domain labeled as R2M1. The protein sequences of these micro‐exons were collected and aligned, and the positions of micro‐exons were marked. Furthermore, the functional sites were predicted by using InterProScan [32]. After alignment correction, KaKs Calculator 2.0 [44, 45] was used to calculate the *K*_a_/*K*_s_ values of these micro‐exons and the AP2 domain with the Yang‐Nielsen approach [29] for determining and comparing their selection in evolution.

For the *MIKC* genes, we first collected all of the 225 K‐box domains from the eight species and identified the motifs of K‐box domains by using MEME. Then the 31 *MIKC* genes in MH63 were used to display the motifs of the K‐box domains. We determined the location of micro‐exons in K‐box domains based on the above information. After that, we appraised the two types of micro‐exon in K‐box domains according to the length and location of micro‐exons. These two kinds of micro‐exon were generally in tandem and labeled as M1 and M2, respectively. Last, the sequences of the micro‐exons and K‐box domains of eight species were collected and aligned, and then the *K*_a_/*K*_s_ values of them were calculated.

## Conflict of interest

The authors declare no conflict of interest.

## Author contributions

QS, AB and HL contributed to the acquisition and analysis of the data, and wrote the draft of the manuscript. L‐LC contributed to the conception and design of the analysis, supervision and analysis of the data, and improving the manuscript. All authors read and approved the final manuscript.

## Supporting information


**Table S1.** The summary of AP2/ERF genes in 63 plants.
**Table S2.** The summary of micro‐exons in AP2 domains and AP2/ERF genes.
**Table S3.** The summary of MIKC genes and micro‐exons in 63 plants.
**Fig. S1.** The summary of MIKC genes and micro‐exons in 63 plants.
**Fig. S2.** The gene structures of MIKC genes in MH63. Thirty‐one MIKC genes are shown.
**Fig. S3.** The gene expressions and domains in MIKC genes via MADS‐box domains.

## Data Availability

The protein sequences and genomic annotations of MH63 and ZS97 are available from the Rice Information Gateway (http://rice.hzau.edu.cn/rice_rs1; version RS1), while the information of other plant species is available in Ensembl Plants (Release 43). RNA‐seq of MH63 and ZS97 are available from the corresponding author upon reasonable request.

## Data accessibility

The protein sequences and genomic annotations of MH63 and ZS97 are available from the Rice Information Gateway (http://rice.hzau.edu.cn/rice_rs1; version RS1), while the information of other plant species is available in Ensembl Plants (Release 43). RNA‐seq of MH63 and ZS97 are available from the corresponding author upon reasonable request.
