# Supplementary material for: Identification and analysis of micro‐exons in AP2/ERF and MADS gene families
Source: FEBS Open Bio. 2020 Nov 8;10(12):2564–77. doi: 10.1002/2211-5463.12990 (PMC7714060; doi:10.1002/2211-5463.12990)
Supplement: Supplementary file 1 — Table S1. The summary of AP2/ERF genes in 63 plants. Table S2. The summary of micro‐exons in AP2 domains and AP2/ERF genes. Table S3. The summary of MIKC genes and micro‐exons in 63 plants. Fig. S1. The summary of MIKC genes and micro‐exons in 63 plants. Fig. S2. The gene structures of MIKC genes in MH63. Thirty‐one MIKC genes are shown. Fig. S3. The gene expressions and domains in MIKC genes via MADS‐box domains. [file FEB4-10-2564-s001.pdf]

Supporting Information

Supplementary Table 1 The summary of AP2/ERF genes in 63 plants.

Species	ERF/DREB	AP2	RAV	Others
Actinidia chinensis	237	32	4	0
Aegilops tauschii	159	23	10	1
Amborella trichopoda	63	8	1	0
Arabidopsis halleri	127	13	6	3
Arabidopsis lyrata	128	13	6	1
Arabidopsis thaliana	125	13	5	0
Beta vulgaris	73	10	3	1
Brachypodium distachyon	132	23	4	2
Brassica napus	454	32	21	10
Brassica oleracea	243	23	9	9
Brassica rapa	242	21	12	8
Chlamydomonas reinhardtii	17	1	0	1
Corchorus capsularis	86	10	2	0
Cucumis sativus	119	16	2	0
Daucus carota	179	18	4	4
Dioscorea rotundata	71	12	1	1
Glycine max	308	40	4	1
Gossypium raimondii	228	27	8	0
Helianthus annuus	258	28	4	2
Hordeum vulgare	134	14	8	0
Leersia perrieri	119	22	4	1
Lupinus angustifolius	200	30	4	0
Manihot esculenta	159	27	7	0
Medicago truncatula	185	24	2	0
Musa acuminata	208	41	14	10
Nicotiana attenuata	174	20	2	4
Oryza barthii	96	15	2	2
Oryza brachyantha	74	17	1	1
Oryza glaberrima	113	20	3	1
Oryza glumipatula	123	20	3	1
Oryza sativa spp. *indica* (93-11)	134	24	4	2
Oryza sativa spp. *indica* (MH63)	113	22	4	2
Oryza sativa spp. *indica* (ZS97)	109	23	3	1
Oryza longistaminata	37	7	0	4
Oryza meridionalis	120	22	4	1
Oryza nivara	133	24	4	2
Oryza punctata	123	25	4	2
Oryza rufipogon	129	22	3	0

Oryza sativa spp. *japonica*	107	19	4	0
Ostreococcus lucimarinus	7	1	0	0
Panicum hallii fil2	142	23	5	0
Panicum hallii hal2	145	25	5	0
Phaseolus vulgaris	153	23	2	1
Physcomitrella patens	137	8	2	6
Populus trichocarpa	177	29	3	1
Prunus persica	104	16	4	1
Selaginella moellendorffii	90	10	4	4
Setaria italica	138	23	4	0
Solanum lycopersicum	142	17	2	7
Solanum tuberosum	184	12	2	2
Sorghum bicolor	147	21	3	0
Theobroma cacao	100	16	3	1
Trifolium pratense	141	15	2	4
Triticum aestivum	476	61	26	3
Triticum dicoccoides	258	38	18	0
Triticum urartu	33	15	2	1
Vigna angularis	163	16	3	1
Vigna radiata	117	12	0	1
Vitis vinifera	114	16	3	0
Zea mays	190	21	5	6

Supplementary Table 2 The summary of micro-exons in AP2 domains and AP2/ERF genes.

Species	Micro-exons in AP2 domains	Micro-exons in genes	Ratio
Actinidia chinensis	59	64	92.2%
Aegilops tauschii	33	46	71.7%
Amborella trichopoda	13	15	86.7%
Arabidopsis halleri	28	32	87.5%
Arabidopsis lyrata	30	73	41.1%
Arabidopsis thaliana	32	37	86.5%
Beta vulgaris	22	22	100.0%
Brachypodium distachyon	43	48	89.6%
Brassica napus	79	121	65.3%
Brassica oleracea	40	53	75.5%
Brassica rapa	31	40	77.5%
Chlamydomonas reinhardtii	4	6	66.7%
Corchorus capsularis	17	22	77.3%
Cucumis sativus	38	41	92.7%
Daucus carota	41	54	75.9%
Dioscorea rotundata	17	24	70.8%
Glycine max	93	104	89.4%
Gossypium raimondii	53	59	89.8%
Helianthus annuus	65	76	85.5%
Hordeum vulgare	24	34	70.6%
Leersia perrieri	22	40	55.0%
Lupinus angustifolius	50	60	83.3%
Manihot esculenta	53	58	91.4%
Medicago truncatula	40	47	85.1%
Musa acuminata	102	135	75.6%
Nicotiana attenuata	46	49	93.9%
Oryza barthii	24	44	54.5%
Oryza brachyantha	33	53	62.3%
Oryza glaberrima	33	41	80.5%
Oryza glumipatula	28	38	73.7%
Oryza sativa spp. *indica* (93-11)	40	49	81.6%
Oryza sativa spp. *indica* (MH63)	40	49	81.6%
Oryza sativa spp. *indica* (ZS97)	41	45	91.1%
Oryza longistaminata	19	45	42.2%
Oryza meridionalis	24	36	66.7%
Oryza nivara	32	44	72.7%
Oryza punctata	31	39	79.5%
Oryza rufipogon	29	43	67.4%
Oryza sativa spp. *japonica*	44	47	93.6%
Panicum hallii fil2	43	46	93.5%
Panicum hallii hal2	46	49	93.9%
Phaseolus vulgaris	54	62	87.1%

Physcomitrella patens	8	13	61.5%
Populus trichocarpa	52	62	83.9%
Prunus persica	33	35	94.3%
Selaginella moellendorffii	14	49	28.6%
Setaria italica	38	45	84.4%
Solanum lycopersicum	40	65	61.5%
Solanum tuberosum	33	36	91.7%
Sorghum bicolor	40	46	87.0%
Theobroma cacao	33	36	91.7%
Trifolium pratense	30	44	68.2%
Triticum aestivum	100	115	87.0%
Triticum dicoccoides	55	88	62.5%
Triticum urartu	17	31	54.8%
Vigna angularis	30	38	78.9%
Vigna radiata	22	44	50.0%
Vitis vinifera	30	43	69.8%
Zea mays	31	41	75.6%

Supplementary Table 3 The summary of MIKC genes and micro-exons in 63 plants.

Species	MIKC genes	Micro-exons in K-box	Micro-exons in MIKC genes
Actinidia chinensis	53	96	98
Aegilops tauschii	37	63	67
Amborella trichopoda	15	28	31
Arabidopsis halleri	29	39	41
Arabidopsis lyrata	34	61	75
Arabidopsis thaliana	38	66	67
Beta vulgaris	26	50	51
Brachypodium distachyon	32	57	59
Brassica napus	138	241	262
Brassica oleracea	63	107	122
Brassica rapa	79	126	131
Corchorus capsularis	7	14	16
Cucumis sativus	6	11	11
Daucus carota	5	9	11
Dioscorea rotundata	19	31	32
Glycine max	85	149	156
Gossypium raimondii	46	81	82
Helianthus annuus	54	97	99
Hordeum vulgare	33	50	56
Leersia perrieri	32	57	69
Lupinus angustifolius	24	36	42
Manihot esculenta	36	68	68
Medicago truncatula	35	68	70
Musa acuminata	54	86	93
Nicotiana attenuata	34	64	64
Oryza barthii	34	56	66
Oryza brachyantha	31	53	76
Oryza glaberrima	29	50	56
Oryza glumipatula	33	58	70
Oryza sativa spp. *indica* (93-11)	31	54	62
Oryza sativa spp. *indica* (MH63)	31	49	52
Oryza sativa spp. *indica* (ZS97)	31	52	55
Oryza longistaminata	25	44	51
Oryza meridionalis	32	59	64
Oryza nivara	30	50	57
Oryza punctata	34	57	67
Oryza rufipogon	34	57	68
Oryza sativa spp. *japonica*	36	59	62
Panicum hallii fil2	37	65	67
Panicum hallii hal2	37	64	68
Phaseolus vulgaris	28	49	51
Physcomitrella patens	6	10	12

Populus trichocarpa	50	97	102
Prunus persica	32	63	66
Selaginella moellendorffii	8	10	14
Setaria italica	22	39	39
Solanum lycopersicum	30	57	61
Solanum tuberosum	28	51	52
Sorghum bicolor	35	63	65
Theobroma cacao	32	58	60
Trifolium pratense	23	37	42
Triticum aestivum	125	207	217
Triticum dicoccoides	60	103	113
Triticum urartu	20	30	32
Vigna angularis	27	45	54
Vigna radiata	24	37	44
Vitis vinifera	28	50	52
Zea mays	45	69	71

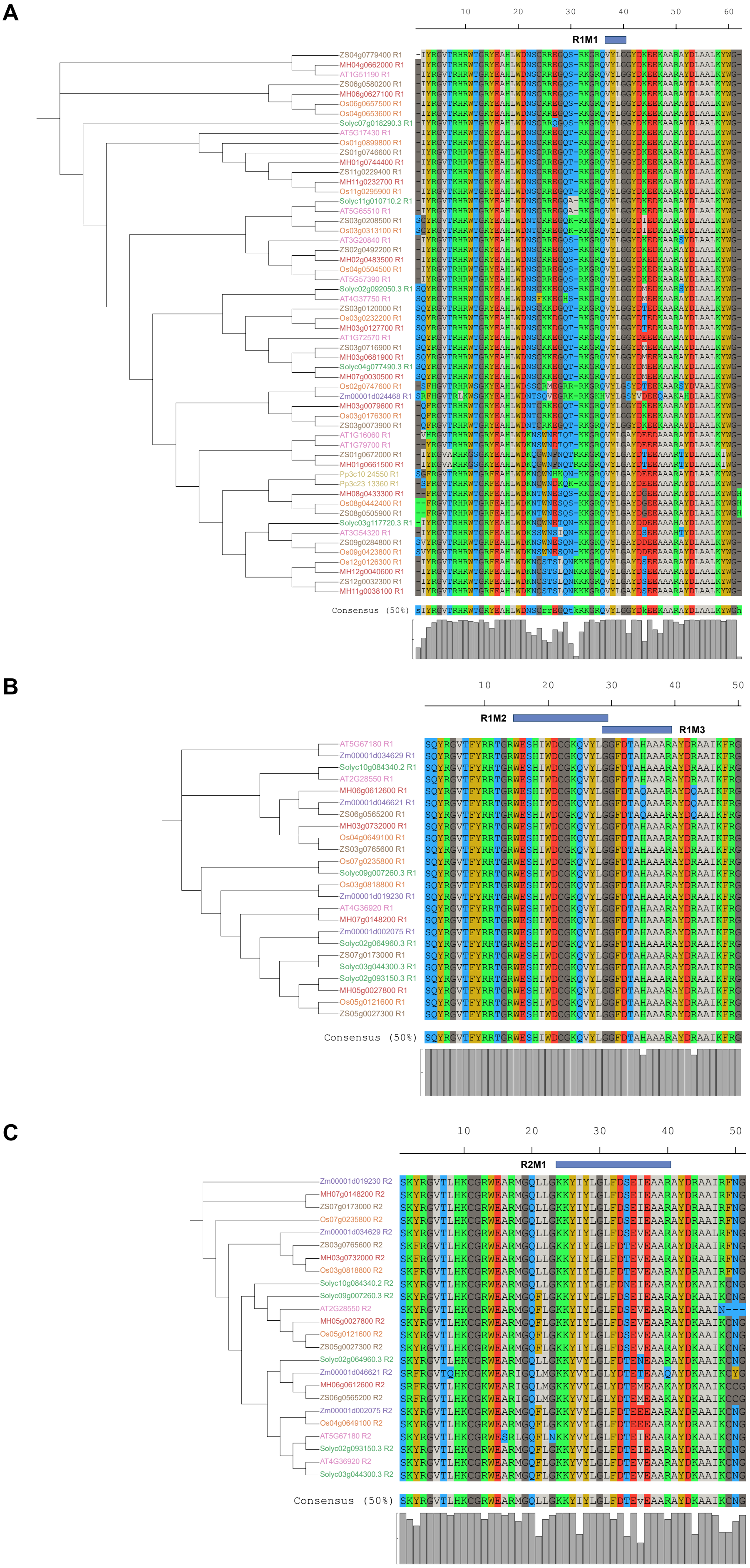

Supplementary Fig. 1 The alignments of micro-exons in AP2 domains among multiple species. Gene from nine species were marked in nine colors; A: The alignment of micro-exons (R1M1) among nine species; B: The alignment of micro-exons (R1M2 and R1M3) among six species; C: The alignment of micro-exons (R2M1) among six species. Sequences encoded by micro-exons were marked above the alignments.

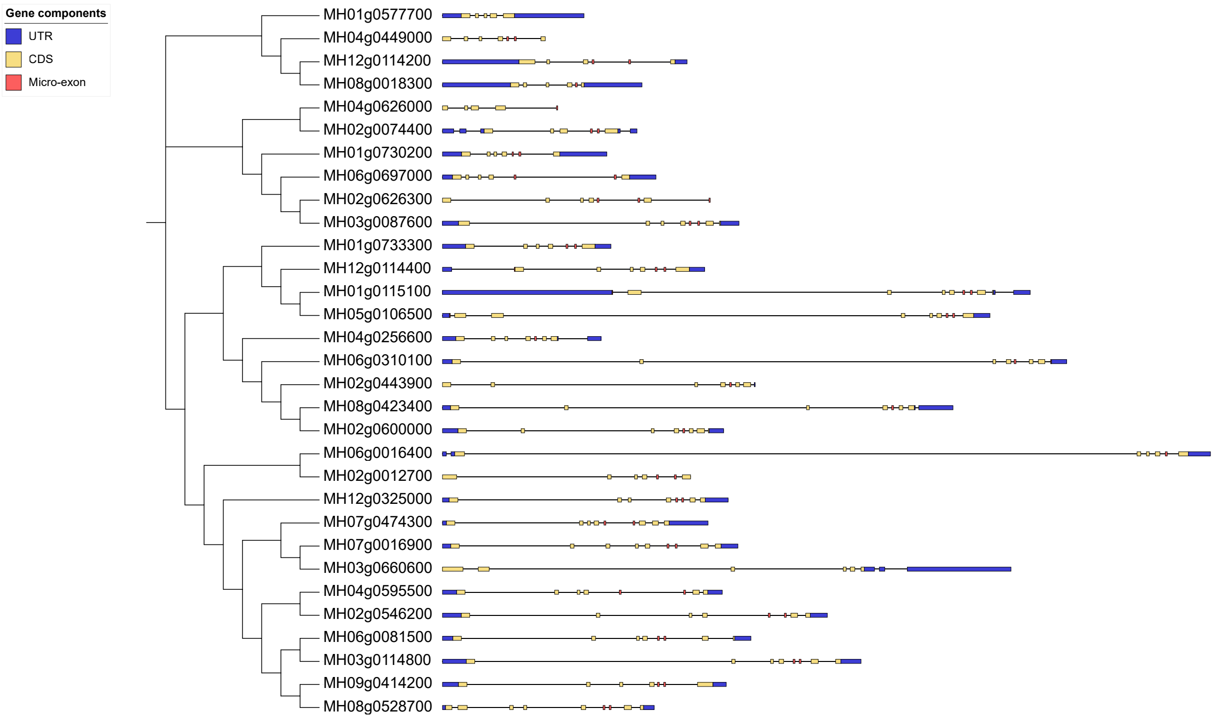

Supplementary Fig. 2 The gene structures of MIKC genes in MH63. Thirty-one MIKC genes are shown. Blue, yellow and red colors demonstrate UTR, CDS and micro-exons of the gene structures, respectively.

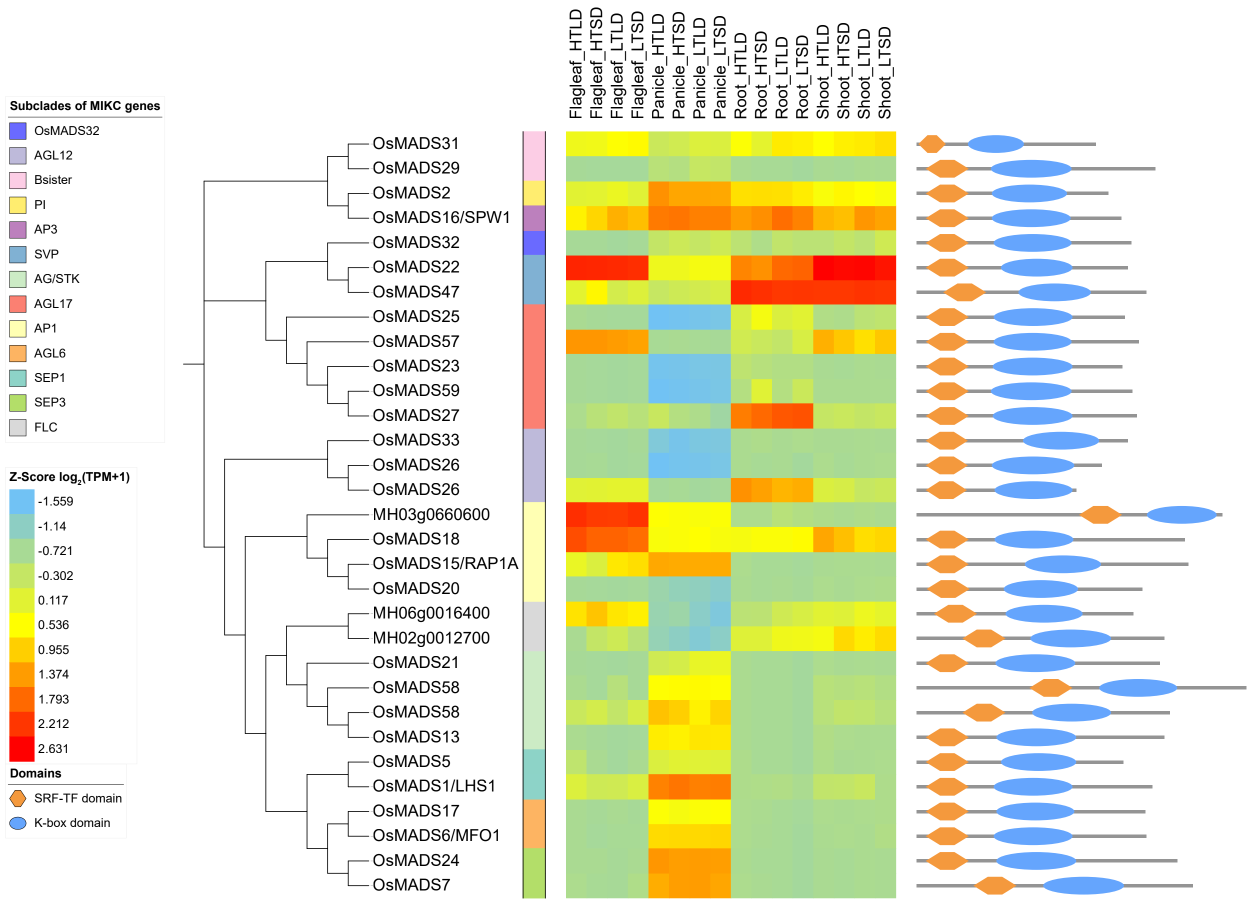

Supplementary Fig. 3 The gene expressions and domains in MIKC genes via MADS-box domains. The left column demonstrates 13 MIKC subfamilies, the middle heatmap represents gene expression levels with Z-score of log2(TPM+1), and the right chart illustrates MADS-box domain and K-box domain in MIKC genes.
